# Emotionally Informed Hate Speech Detection: A Multi-target Perspective

**DOI:** 10.1007/s12559-021-09862-5

**Published:** 2021-06-28

**Authors:** Patricia Chiril, Endang Wahyu Pamungkas, Farah Benamara, Véronique Moriceau, Viviana Patti

**Affiliations:** 1 IRIT, Université de Toulouse, Université Toulouse III - UPS, Toulouse, France; 2 Dipartimento di Informatica, University of Turin, Turin, Italy

**Keywords:** Hate speech detection, Hate speech targets, Affective resources, Multi-task learning, Social media

## Abstract

Hate Speech and harassment are widespread in online communication, due to users' freedom and anonymity and the lack of regulation provided by social media platforms. Hate speech is topically focused (misogyny, sexism, racism, xenophobia, homophobia, etc.), and each specific manifestation of hate speech targets different vulnerable groups based on characteristics such as gender (misogyny, sexism), ethnicity, race, religion (xenophobia, racism, Islamophobia), sexual orientation (homophobia), and so on. Most automatic hate speech detection approaches cast the problem into a binary classification task without addressing either the *topical focus* or the *target-oriented* nature of hate speech. In this paper, we propose to tackle, for the first time, hate speech detection from a multi-target perspective. We leverage manually annotated datasets, to investigate the problem of transferring knowledge from different datasets with different topical focuses and targets. Our contribution is threefold: (1) we explore the ability of hate speech detection models to capture common properties from topic-generic datasets and transfer this knowledge to recognize specific manifestations of hate speech; (2) we experiment with the development of models to detect both topics (racism, xenophobia, sexism, misogyny) and hate speech targets, going beyond standard binary classification, to investigate *how to detect hate speech at a finer level of granularity* and *how to transfer knowledge across different topics and targets*; and (3) we study the impact of affective knowledge encoded in sentic computing resources (SenticNet, EmoSenticNet) and in semantically structured hate lexicons (HurtLex) in determining specific manifestations of hate speech. We experimented with different neural models including multitask approaches. Our study shows that: (1) training a model on a combination of several (training sets from several) topic-specific datasets is more effective than training a model on a topic-generic dataset; (2) the multi-task approach outperforms a single-task model when detecting both the hatefulness of a tweet and its topical focus in the context of a multi-label classification approach; and (3) the models incorporating EmoSenticNet emotions, the first level emotions of SenticNet, a blend of SenticNet and EmoSenticNet emotions or affective features based on Hurtlex, obtained the best results. Our results demonstrate that multi-target hate speech detection from existing datasets is feasible, which is a first step towards hate speech detection for a specific topic/target when dedicated annotated data are missing. Moreover, we prove that domain-independent affective knowledge, injected into our models, helps finer-grained hate speech detection.

## Introduction

Nowadays, people increasingly use social networking sites, not only as their main source of information, but also as media to post content, sharing their feelings and opinions. Social media is convenient, as sites allow users to reach people worldwide, which could potentially facilitate a positive and constructive conversation between users. However, this phenomenon has a downside, as there are more and more episodes of hate speech (HS hereafter) and harassment in online communication [10]. This is due especially to the freedom and anonymity given to users and to the lack of effective regulations provided by the social network platforms. There has been a growing interest in using artificial intelligence and Natural Language Processing (NLP) to address social and ethical issues. Let us mention the latest trends on *AI for social good* [40, 41], where the emphasis is on developing applications to maximize “good” social impacts while minimizing the likelihood of harm and disparagement to those belonging to vulnerable categories. See, for example, the literature on suicidal ideation detection, devoted to early intervention [48]. There are also recent works on the prevention of sexual harassment [68], sexual discrimination [67], cyberbullying and trolling [81], devoted to contrasting different kinds of abusive behavior targeting different groups and preventing unfair discrimination.

In spite of there being no universally accepted definition of HS, this study employs the most common one. HS is defined here as any type of communication that is abusive, insulting, intimidating, and/or that incites violence or discrimination, and that disparages a person or a vulnerable group based on characteristics such as ethnicity, gender, sexual orientation and religion [33]. Accordingly, HS may have different topical focuses: misogyny, sexism, racism, xenophobia and homophobia or Islamophobia, which we refer to as **topics**. For each topic, hateful content is directed towards specific **targets** that represent the community (individuals or groups) receiving the hatred. For example, black people and white people are possible targets when the topical focus is *racism* [117], while women are the targets when the topical focus is *misogyny* or *sexism* [78]. HS is thus, by definition, *target-oriented*, as shown in the following tweets taken from [5, 25, 133], where the targets are underlined. These examples also show that different targets involve different ways of linguistically expressing hateful content such as references to racial or sexist stereotypes, the use of negative and positive emotions, swearing terms, and the presence of other phenomena such as envy and ugliness.^1^

1. *Women who are feminist are the ugly bitches who cant find a man for themselves*
2. *Islam is 1000 years of contributing nothing to mankind but murder and hatred.*
3. *Illegals are dumping their kids heres o they can get welfare, aid and U.S School Ripping off U.S Taxpayers #SendThemBack ! Stop Allowing illegals to Abuse the Taxpayer #Immigration*
4. *Seattle Mayoral Election this year. A choice between a bunch of women, non-whites,* and *faggots/fag lovers.*

Given the vast amount of social media data produced every minute^2^, manually monitoring social media content is impossible. It is, instead, necessary to detect HS automatically. To this end, many studies in the field exploit supervised approaches generally casting HS detection as a binary classification problem (i.e., abusive/hateful vs. not abusive/not hateful) [43, 64, 115] relying on several manually annotated datasets that can be grouped into one of these categories:

- *Topic-generic* datasets, with a broad range of HS without limiting it to specific targets [21, 44, 52]. For example, [21] consider aggressive and bullying in their annotation scheme, while [44] looks, in addition, for other expressions of online abuse such as offensive, abusive and hateful speech.
- *Topic-specific* datasets, where the HS category (racism, sexism, etc.) is known in advance (i.e., drives the data gathering process) and is often labeled. The HS targets, either person-directed or group-directed^3^, can be considered as *oriented*, containing, as they do, hateful content towards groups of targets or specific targets. For example, in [132] scholars sampled data for multiple targets, that is racism and sexism for, respectively, religious/ethnic minorities HS and sexual/gender (male and female) HS. Others focus on single targets including, for instance, sampling for the misogyny topic, targeting women [23, 38, 39]. Similarly, for the xenophobia and racism topics the target are groups discriminated against on the grounds of ethnicity (e.g., immigrants [5], ethnic minorities [125, 133], religious communities [128], Jewish communities [145], etc.).

Independently from the datasets that are used, all existing systems share two common characteristics. First, they are trained to predict the presence of general, target-independent HS, without addressing the problem of the variety of aspects related to both the topical focus and target-oriented nature of HS. Second, systems are built, optimized, and evaluated based on a single dataset, one that is either topic-generic or topic-specific. In order to address this issue and in order to improve the performance of the models, recent studies propose cross-domain classification, where the domain is used synonymously with dataset [65, 99, 134, 137]. The idea consists in using a one-to-one configuration by training a system on a given dataset and testing the system on another one, using domain adaptation techniques. Most existing works map between fine-grained schemes (that are specific for each dataset) and a unified set of tags, usually composed of a positive and negative label to account for the heterogeneity of labels across datasets. Again, this binarization fails to discriminate among the multiple HS targets. Thus, it has become difficult to measure the generalization power of such systems and, more specifically, their ability to adapt their predictions in the presence of novel or different topics and targets [126].

An immediate but rather expensive solution for handling a new specific target is that of building new target-oriented datasets from scratch; as has been done in previous studies [61]. In this paper, we propose instead a novel multi-target HS detection approach by leveraging existing manually annotated datasets. These will enable the model to transfer knowledge from different datasets with different topics and targets. In the context of offensive content moderation, identifying the topical focus and the targeted community of hateful contents would be of great interest for two important reasons. First, it will allow us to detect HS for specific topics/targets when dedicated data are missing. Second, it will prevent widespread stereotypes and help to develop social policies for protecting victims, especially in response to trigger events [69]. For example, with the recent outbreak of COVID-19, a spike in racist and xenophobic messages targeting Asians in Western countries was observed. A system specifically designed to detect HS that targets migrants in a pre-COVID-19 context would most likely have failed at picking out this post-COVID-19 HS. Indeed, most of the messages would not have been moderated as the type of language learned during training was for other groups, the most frequent targets of HS in pre-COVID times.

In this paper, we consider different manifestations of HS with different topical focuses, including sexism, misogyny, racism, and xenophobia. Each specific instance targets different vulnerable groups based on characteristics such as gender (sexism and misogyny), ethnicity, religion and race (xenophobia and racism). The focus on gendered and ethnicity-based HS is due, in part, to the wide availability of English corpora developed by the computational linguistics community for those targets. But it also depends on the fact that most monitoring exercises by institutions countering online HS in different countries and territories (e.g., European Commission [34]) report ethnic-based hatred (including anti-migrant hatred) and gender-based hatred as the most common type of online HS [22]. We propose to undertake the following challenges:

1. **Explore the ability of HS detection models to capture common properties from generic HS datasets and to transfer this knowledge to recognize specific manifestations of hate**. We propose several deep learning models and experiment with binary classification using two generic corpora. We evaluate their ability to detect HS in four topically focused datasets: sexism, misogyny, racism, and xenophobia. Our results show that training on topic-generic datasets generally fails to account for topic-specific linguistic properties.
2. **Experiment with the development of models for detecting both the topics (racism, xenophobia, sexism, misogyny) and the targets (gender, ethnicity) of HS** going beyond standard binary classification. We aim to investigate (a) *how to detect HS at a finer level of granularity* and (b) *how to transfer knowledge across different types of HS*. We rely on multiple topic-specific datasets and develop, in addition to the deep learning models designed to address the first challenge, a multitask architecture that has been shown to be quite effective in cross-domain sentiment analysis [12, 146]. We consider several experimental scenarios: first, ones where the topics/targets that will be classified in a multi-label fashion are present in the training data; and second, in cross-topic/target scenarios, where we try to predict a specific target/topic, training on data where that particular topic/target is unseen. Our results demonstrate that learning HS classification (main task) and the topic/target of HS (auxiliary task) simultaneously achieves very good results. This result is an encouraging first step, demonstrating that multi-target HS detection from existing datasets is feasible. This is true even in the absence of target-specific data towards a given target, something which can be of crucial importance when annotated data about the target are missing.
3. **Study the impact of affective semantic resources in determining specific manifestations of HS**. Affects and emotions were proven to be useful in many NLP tasks such as irony and sarcasm detection [57, 98, 120], stance classification [71, 72], information credibility assessment [49, 50], and also sentiment analysis [20, 76] in general. In this work, we also want to explore the affective characteristics of the language used in HS, continuing the very recent work by [109], which suggests a strong relationship between abusive behavior and the emotional state of the speaker. We experiment with three affect resources as extra-features on top of several deep learning architectures: sentic computing [14] resources (SenticNet [18], EmoSenticNet [106]) and semantically structured hate lexicons (HurtLex [6]). SenticNet has not, to the best of our knowledge, been used in HS detection. For each resource, we propose a systematic evaluation of the emotional categories that are the most productive for our tasks. Our results show that injecting domain-independent affective knowledge into our models helps finer-grained HS detection.

The remainder of this paper is organized as follows. In the next section, we present an overview of the main works on HS detection. Datasets describes the datasets used in this study. Generalizing Hate Speech Phenomena Across Multiple Datasets, Multi-target Hate Speech Detection, Emotion-aware Multi-target Hate Speech Detection detail, respectively: the experiments carried out and the results obtained when generalizing HS phenomena across multiple datasets; predicting multi-target HS; and building emotionally informed models. We end this paper by discussing our main findings and by providing directions for future work.

## Related Work

We present the related work in four parts. First, we briefly introduce the affective computing and sentiment analysis research field, in order to provide readers with a broader context for NLP literature related to the analysis and to the recognition of affective states and emotions in texts. Second, relevant prior works specifically related to HS detection are presented. Third, we review the domain adaptation study in sentiment analysis and abusive language detection, something particularly important in bringing out the novelty of our contribution. Finally, we provide an overview of the few attempts to exploit affective information in improving abusive language detection.

### Affective Computing and Sentiment Analysis

Affective computing, a development of the last decades, is the study and development of systems and devices that can recognize, interpret, process, and simulate human affects: i.e., the experience of feelings or emotions. Today, identifying affective states from text is regarded as being fundamental for several domains, from human-computer interaction to artificial intelligence, from the social sciences to software engineering [13]. The wide popularity of social media, which facilitates users publishing and sharing contents—providing accessible ways for expressing feelings and opinions about anything, anytime—also gave a major boost to this research area. This was especially true within the NLP field. Here, the abundance of data allowed the research community to tackle more in-depth, long-standing questions such as understanding, measuring and monitoring the sentiment of users towards certain topics or events, expressed in mere texts or through visual and vocal modalities [107]. Indeed, robust and effective approaches are made possible by the rapid progress in supervised learning technologies and the huge amount of user-generated content available online. Such techniques are typically motivated by the need to extract user opinions on a given product or, say, in surveying political views and they often exploit knowledge encoded in affective resources, such as sentiment and emotion lexicons and ontologies.

The interest in lexical knowledge about the multi-faceted and the fine-grained facets of affect encoded in such resources is, by no means, limited to sentiment analysis. The use of such affective resources has also recently been explored in other related tasks, such as personality [80, 86] and irony detection [35, 120] or author profiling [100]. Concerning abusive language detection, which is the specific task of interest here, there are attempts at exploiting emotion signals to improve the detection of this kind of phenomena (cf. Affective Information in Abusive Language Detection Tasks). No one has investigated the impact of emotion features on HS detection, which is one of the challenges tackled in our paper.

#### Supervised and Semi-Supervised Learning for Social Data Analysis

The field has recently been surveyed in [7, 142]. The vast majority of the analyzed papers describe approaches to sentiment analysis based on supervised learning, where there is a text classification task at the sentence or message level, focused mostly on detecting from text valence or *sentiment*, either using a binary value or with a strength/intensity component coupled with the sentiment [123]. In particular, deep learning-based methods are becoming very popular due to their high performance, and they have been increasingly applied in sentiment analysis [82, 142]. Furthermore, there is an ever-increasing awareness of the need to take a holistic approach to sentiment analysis [17] by handling the many finer-grained tasks involved in extracting meaning, polarity and specific emotions from texts. This includes the detection of irony and sarcasm [57, 66, 120].

Due to a large amount of available (but unlabeled) data, many studies have recently highlighted the importance of exploring unsupervised and semi-supervised machine learning techniques for sentiment analysis tasks. For example in [60], the authors exploited both labeled and unlabeled commonsense data. Their proposed affective reasoning architecture is based on Support Vector Machines (SVM) and the merged use of random projection scaling in a vector space model and was exploited for emotion recognition tasks.

#### Emotion Categorization Models and Affective Resources

Still, despite the maturity of the field, choosing the right model for operationalizing affective states is not a trivial task. Research in sensing sentiment from texts has put the major emphasis on recognizing polarities (positive, negative, neutral orientation). However, comments and opinions are usually directed toward a specific target or aspect of interest, and as such, finer-grained tasks can be envisioned. For instance, aspect-based sentiment analysis identifies the aspects of given target entities and the sentiment expressed for each aspect [105]. At the same time, the stance detection emerging task focuses on detecting what particular stance a user takes toward a specific target, something that is particularly interesting in political debates [89].

Moreover, given the wide variety of affective states, recent studies advocate a finer-grained investigation of the role of *emotions*, as well as the importance of other affect dimensions such as emotional intensity or activation. Depending on the specific research goals addressed, one might be interested in issuing a discrete label describing the affective state expressed (frustration, anger, joy, etc.) in accordance with different contexts of interaction and tasks. Emotions are transient and typically episodic, in the sense that, over time, they can come and go. This depends, of course, on all sorts of factors, factors which researchers might be interested in understanding and modeling according to a domain or task-specific research objectives.

Both basic emotion theories, in the Plutchik-Ekman tradition [32, 104], and dimensional models of emotions [112] provide a precious theoretical grounding for the development of lexical resources and computational models for affect extraction. Sentiment-related information is, indeed, often encoded in lexical resources, such as affective lists and corpora, where different nuances of affect are captured, such as sentiment polarity, emotional categories, and emotional dimensions [18, 90, 106]. These kinds of lexicons are usually lists of words to which a positive or negative or/and an emotion-related label (or score) is associated. Besides flat lists of affective words, lexical taxonomies have also been proposed, enriched with sentiment and/or emotion information [3, 106]. However, there is a general tendency to go towards richer, finer-grained models. These will very possibly include complex emotions. This is especially the case in the context of data-driven and task-driven approaches, where restricting automatic detection to only a small set of basic emotions is too limited, not least in terms of actionable affective knowledge. This general tendency is also reflected in the development of semantically richer resources. These include and model semantic, conceptual, and affective information associated with multi-word natural language expressions, by enabling the concept-level analysis of sentiment and emotions conveyed in texts, like the ones belonging to the SenticNet family [15, 18]. Moreover, when the task addressed is related to a specific portion of the affective space, domain-specific affective resources and lexicons can be envisioned. This is the case with abusive language detection, where the use of lexicons of hateful words [6] can lead to interesting results.

#### Word Intensity and Polarity Disambiguation

All such resources represent a rich and varied lexical knowledge about affect, under different perspectives, and virtually all sentiment analysis systems may incorporate lexical information derived from them^4^. However, many opinion keywords carry varying polarities in different contexts, posing huge challenges for sentiment analysis research. Contextual polarity ambiguity is an important still little studied problem in sentiment analysis. This has recently been addressed in [140], where a Bayesian model is proposed that uses opinion-level features to solve the polarity problem of sentiment-ambiguous words: intra-opinion features (i.e., the information that helps in thoroughly conveying the opinion); and inter-opinion features (i.e., the information connecting two or more opinions). The intra-opinion features resolve the polarity of most sentiment words. The inter-opinion features usually play a secondary role, either by improving the confidence of a good prediction or by assisting in calculations when some of the features are missing.

Another interesting challenge for the field is related to the possibility of measuring sentiment and emotion intensity, which is of paramount importance in analyzing the finer-level details of emotions and sentiments [85] in real-world applications. A novel solution to this problem is proposed in [2], where, in order to leverage the various advantages of different supervised systems, a Multi-Layer Perceptron (MLP)-based ensemble framework for predicting the intensity of sentiments (in financial microblog messages and news headlines) and emotions (in tweets) is proposed. The ensemble model combines the output of three deep learning models (Convolutional Neural Network (CNN), Long Short-Term Memory (LSTM), and Gated Recurrent Unit (GRU)) and a feature-based Support Vector Regression (SVR) model. The SVR model utilizes word and character TF-IDF, TF-IDF weighted word vectors, and a diverse set of lexicon features, such as the positive and negative word count (extracted from MPQA [135] and Bing Liu [29]), the positive, negative, and aggregate scores of each word extracted from NRC Hashtag Sentiment and NRC Sentiment140 [88], as well as the sum of the positive, negative and aggregate scores of each word computed from SentiWordNet [3]. For emotion intensity prediction, the authors also include: the word count of each of the emotions from NRC Word-Emotion Association lexicon [87]; the sum of association scores for the words with the emotions extracted from NRC Hashtag Emotion [84]; the aggregate of positive and negative word scores computed from AFINN [94]; and the sentiment score of each sentence returned by VADER [51]. The proposed framework shows good results with comparatively better performance over state-of-the-art systems.

### Hate Speech Detection in Online Communication

The automatic detection of online HS is not a simple task, especially because of the thin line between abusive language and freedom of speech. For example, the use of swear words could become an issue in HS detection [96, 122], where their presence might lead to false positives: for instance, when they are used in a non-abusive way in humor, emphasis, catharsis, and when conveying informality. But they could also become a strong signal for spotting HS, when they are used in an abusive context.

Most studies that deal with automatic HS detection exploit supervised approaches to classify HS and non-HS content. First studies in the field relied on traditional machine learning approaches with hard-coded features. Several classifiers were used, such as Logistic Regression (LR) [4, 26, 30, 36, 83, 133], SVM [4, 9–11, 55, 124, 131], Naive Bayes (NB) [1, 70], Decision Tree (DT) [1, 9–11], and Random Forest (RF) [1, 4, 9–11]. A wide range of features have been employed including lexical features (e.g., n-grams, Bag of Words, TF-IDF, lexicon-based); syntactic features (e.g., speech parts and typed dependency); stylistic features (e.g., number of characters, punctuation, text length); as well as some Twitter specific features (e.g., the number of user mentions, hashtags, URLs, social network information [83]; and other user features [36, 108, 133]). Recently, the task of automatic HS detection has focused on exploiting neural models such as LSTM [83, 129], Bidirectional Long Short-Term Memory (Bi-LSTM) [108], GRU [91], and CNN [4] coupled with word embedding models such as FastText^5^, word2vec^6^, and ELMo [103].

A fair amount of works that deals with HS detection have come from teams that participated in recently shared tasks such as HatEval [5], Automatic Misogyny Identification (AMI) [38, 39], and Hate Speech and Offensive Content Identification (HASOC) [77]. HatEval was introduced at SemEval 2019 and focused on the detection of hateful messages on Twitter directed towards two specific targets: immigrants and women. This was done from a multilingual^7^ perspective (English and Spanish). The best-performing system in English HatEval [62] exploited a straightforward SVM with a Radial Basis Function (RBF) kernel that uses Google’s Universal Sentence Encoder [19] feature representation. AMI, another shared task in two different evaluation campaigns in 2018 (IberEval and Evalita^8^), focuses on detecting HS that targets women. In English, the best results were achieved by traditional models for both AMI-IberEval (SVM with several handcrafted features [97]) and AMI-Evalita (LR coupled with vector representation that concatenates sentence embedding, TF-IDF and average word embeddings [113]). Finally, HASOC, an HS and offensive language identification shared task at FIRE 2019, covers three languages: English, German, and Hindi. For English, the best performance was achieved by an LSTM network with ordered neurons and an attention mechanism [130]. All the aforementioned shared tasks provided datasets in languages other than English: i.e., Italian, Spanish, Hindi, and German. Other languages used in shared tasks include Italian (HasSpeeDe [8] which focuses on detecting HS towards immigrants) and German (GermEval [138] which focuses on offensive language identification).

Most of the works listed here model their tasks as a binary classification, with the aim of predicting the abusiveness of a given utterance *per se* (i.e., without specifying either a topic or a target). In this work, we classify a message as hateful or not-hateful. But we go further. We want also to detect the HS topic and the target to whom the message is addressed. To the best of our knowledge, we are the first to address target-based computational HS detection, continuing recent corpus-based linguistic studies on categorizing HS and their associated targets [117].

### Domain Adaptation in Abusive Language Detection

The study of HS detection is multifaceted, and available datasets feature different focuses and targets. Despite limitations, some works have tried to bridge this range by proposing a domain adaptation approach to transfer knowledge from one dataset to other datasets with different topical focuses.

The first attempt to deal with this issue was reported in [134]. They used the multi-task learning (MTL) approach, arguing that it would be possible to share knowledge between two or more objective functions to leverage information encoded in one abusive language dataset to better-fit others. [65] proposed using a traditional machine learning approach for classifying abusive language in a cross-domain setting, in order to get better system interpretability. This work also explored the use of the *frustratingly simple domain adaptation* (FEDA) framework [24] to facilitate domain sharing between different datasets. The main finding of this work is that the model did not generalize well when applied to various domains, even when trained on a much bigger out-domain dataset. [111] adopted transfer learning as a domain adaptation approach by exploiting the LSTM network coupled with ELMo embeddings. LSTM has also been used by [99], who employed it with a list of abusive keywords from the Hurtlex lexicon [6], as a proxy for transferring knowledge across different datasets. Their main findings are: (i) that the model trained on more than one general abusive language dataset will produce more robust predictions; and (ii) that HurtLex is able to boost the system performance in the cross-domain setting.

Bidirectional Encoder Representations from Transformers (BERT) [28] was also applied in cross-domain abusive language detection [122]. This work found that BERT can share knowledge between one domain dataset and other domains, in the context of transfer learning. They argue that the main difficulty in the cross-domain classification of abusive language is caused by dataset issues and their biases. It is consequently impossible for datasets to capture the phenomenon of abusive language in its entirety. [92] also investigated BERT by using new fine-tuning methods based on transfer learning, relying on Waseem [133] and Davidson [26] datasets in their experiments. Finally, HatEval, a recently shared task [5], also provided an HS dataset that covers two different targets, women and immigrants. Therefore, participants are required to build a target-agnostic model able to detect HS with more than one target (cf. Hate Speech Detection in Online Communication).

Cross-domain classification approaches in abusive language detection share three common characteristics: (1) Dataset labels are aligned to deal with the varieties of annotation schemes. Hence, all datasets (be they topic-generic or topic-specific) share the same coarse-grained characterization of HS (i.e., hateful vs. non-hateful). (2) Systems follow a one-to-one configuration (i.e., they are trained on one dataset and tested on another) in order to analyze their robustness in generalizing the different phenomena contained in each dataset. (3) Predictions are binary, ignoring the target/topic nature of HS. In this work, we intend to focus on the different topics/targets in several datasets by proposing a multi-target HS classification task.

To this end, instead of using the typical one-to-one configuration, we propose to solve the problem using a many-to-many configuration capable of identifying a given topic/target when trained in topic-generic or topic-specific datasets. The many-to-many configuration has already been shown to be quite effective in cross-domain aspect-based sentiment analysis [12, 46, 53, 74, 102, 146] and is used here for the first time in an HS detection task.

### Affective Information in Abusive Language Detection Tasks

Recently, some works exploiting emotion signals to improve abusive language detection have been carried out. The study by [114] proposed an architecture that uses the Emotion-Aware Attention (EA) mechanism to quantify the importance of each word based on the emotion conveyed by the text. They used DeepMoji model [37] and NRC Emotion Lexicon [87] to extract emotion information from the given texts. Their analysis of the results shows the importance of affective information in augmenting system performance. Similar conclusions have been drawn in [96] who exploited the NRC Emotion Lexicon [87] and EmoSenticNet [106]. Finally, the most recent work by [109] came up with a joint model of emotion and abusive language detection in a MTL setting. This led to significant improvements in abuse detection performance when evaluated in both the OffensEval 2019 [144] and Waseem and Hovy datasets [133].

As far as we know, no previous work has explored the impact of emotion features in predicting HS targets in a multi-target setting. We propose to employ EmoSenticNet, HurtLex, and for the first time, SenticNet. For each resource, we identify the emotion categories that are the most suitable for predicting a given topic/target of HS detection.

## Datasets

We experiment with seven available HS corpora from previous studies among which two are topic-generic (**Davidson** [26] and **Founta** [44]), and four are topic-specific about four different topics: *misogyny* (the AMI dataset collection from both **IberEval** [39] and **Evalita** [38]), *misogyny and xenophobia* (the **HatEval** dataset [5]), and *racism* and *sexism* (the **Waseem** dataset [133]). Each of these topics target either gender (sexism and misogyny) and/or ethnicity, religion or race (xenophobia and racism).

In this section, we first detail the characteristics of each of the seven datasets, then provide general statistics.

### Datasets Description

- **Davidson.** The dataset has been built by [26] and contains 24,783 tweets^9^ manually annotated with three labels including *hate speech*, *offensive*, and *neither*. These tweets were sampled from a collection of 85.4 million tweets gathered using the Twitter search API, focusing on tweets containing keywords from HateBase^10^. The dataset was manually labeled by using the CrowdFlower platforms^11^, where at least three annotators annotated each tweet. With an inter-annotator agreement of 92%, the final label for each instance was assigned according to a majority vote. Only 5.8% of the total tweets were labeled as *hate speech* (cf. (5)) and 77.4% as *offensive* (cf. (6)), while the remaining 16.8% were labeled as *not offensive*.
  (5) *#DTLA is trash because of non-Europeans are allowed to live there*
  (6) *What would y’all lil ugly bald headed bitches do if they stop making make-up & weave?*
- **Founta**. The dataset consists of 80,000 tweets^12^ annotated with four mutually exclusive labels including *abusive*, *hateful*, *spam* and *normal* [44]. The original corpus of 30 millions tweets was collected from 30 March 2017 to 9 April 2017 by using the Twitter Stream API. For each tweet, the authors also extracted the meta-information and linguistic features in order to facilitate the filtering and sampling process. Annotation was done by five crowdworkers and the final dataset was composed of 11% tweets labeled as *abusive* (cf. (7)), 7.5% as *hateful* (cf. (8)), 59% as *normal*, and 22.5% as *spam* (cf. (9)).
  (7) *Benedict Cumberbatch is a damn stupid name. I hope history doesn’t remember him fondly. I hope his legacy becomes trash.*
  (8) *Niggas worst than your side bitch always questioning they position*
  (9) *Beats by Dr. Dre urBeats Wired In-Ear Headphones - White https://t.co/9tREpqfyW4 https://t.co/FCaWyWRbpE*
- **Waseem**. It consists of tweets collected over a period of two months by using representative keywords (common slurs) that target religious, sexual, gender and ethnic minorities [133]. The authors manually annotated the dataset with a third expert annotator reviewing their annotations. The final dataset consists of 16,914 tweets, with 3,383 instances from **Sexism**_**Waseem**_ targeting gender minorities (cf.(10)), 1,972 from **Racism**_**Waseem**_ with racist instances (cf. (11)), and 11,559 tweets that were judged to be neither sexist nor racist^13^.
  (10) * Sounds like we’ve got a well good ref’ today, bloody women should just stay in the kitchen!*
  (11) * It’s not about any specific individuals, but about an ideology that will always produce terrorists*.
- **AMI corpora.** The main goal of the AMI task consists in identifying tweets that convey hate or prejudice against women while categorizing forms of misogynous behavior (stereotype & objectification, dominance, derailing, sexual harassment & threats of violence, discredit), as well as classifying the target of a given instance (specific individual or a generic group). The datasets used in these tasks were collected by employing three different approaches: representative keywords and hashtags; monitoring potential victims; as well as by downloading the history of users that have explicitly misogynistic behavior on their Twitter profiles. We use in this study the two AMI datasets: **IberEval** [39] containing 3,977 tweets collected over a period of four months (from 20th of July until 30th of November 2017) and **Evalita**  [38] that comprises 5,000 tweets. Below are two examples of tweets annotated as misogyny taken, respectively, from **IberEval** and **Evalita**. Their associated misogynisitic behavior are ”sexual harassment” in the first example and ”derailing” in the second.
  (12) * I kinda want to see you again just so I can punch you in the kidney. #WomenSuck*
  (13) * Yes yes Ann, lets continue to perpetuate the hysterical woman stereotype. Such a shame. You dont deserve your position of power. A disgrace to fellow women.*
- **HatEval**. The dataset consists of 13,000 tweets distributed across two different targets: immigrants (cf. (14)) and women (cf. (15)) [5]. Most of the tweets that target women were derived from the **AMI corpora**, while the remainder of the dataset was collected over a period of three months (from July to September 2018) by employing the same approaches as AMI. The dataset was annotated by using the Figure Eight crowdsourcing platform. In each instance, the annotators were asked to specify whether a tweet conveys HS or not towards any given targets. The annotators were also asked to indicate whether the author of the tweet was aggressive and to identify the target of the tweet (i.e., a specific individual or a group of people). Although the inter-annotator agreement obtained for each category (0.83, 0.73, and 0.70, respectively) was quite high, the final label was assigned based on a majority vote by adding two expert annotations to the crowd-annotated data. The final distribution of the dataset includes 13,000 tweets (6,500 for each target).
  (14) * Your boats shall drown in the Mediterranean Sea and the rest of you, which had not assimilated into our society will leave immediately. #RefugeesNotWelcome #IllegalAliens*
  (15) * Its a good thing I always wear a glove on my left hand because if I EVER had to touch hands with a woman my IQ would totally drop to 0 Lol*

### Datasets Statistics

Table 1 provides a general overview of the datasets, along with the labels used in their annotation schemes. We can observe that the classes are imbalanced in most datasets, where the majority class is the negative class (non-HS), except for the AMI collection (**AMI-IberEval** and **AMI-Evalita**) and **Davidson**.

**Table 1:** General overview of the datasets along with their topics and targets

Dataset	Labels	# of instances		Topic	Target
**Davidson**	hate speech	1,430	24,783	generic	none
	offensive	19,190			
	neither	4,163			
**Founta**	abusive	27,037	99,799	generic	none
	hateful	4,948			
	spam	14,024			
	normal	53,790			
**Waseem**	racism	1,957	16,488	specific	race, gender
	sexism	3,216			
	none	11,315			
**Evalita**	misogyny	2,245	5,000	specific	women
	not misogyny	2,755			
**IberEval**	misogyny	1,851	3,977	specific	women
	not misogyny	2,126			
**HatEval**	immigrant	2,427	11,971	specific	women, ethnicity
	women	2,608			
	not hate speech	6,936			

For our experiments, the corpora have been divided into train and test sets keeping the same tweet distribution as the original papers. This was done in order to make better comparisons with the state-of-the-art results^14^. Table 2 and Table 3 provide the distribution of instances in these two sets. As one of the research questions that we want to address involves the possibility of transferring knowledge from several topic-specific datasets into another topic-specific dataset where the topic is unseen, we decided to merge under the same topic (i.e., misogyny) both the **AMI corpora** and **HatEval** **dataset**^15^.

**Table 2:** Distribution of instances in topic-generic datasets (used as training)

Dataset	Labels	# of instances	
**Founta**	hateful	1,930	39,700
	not-hateful	37,770	
**Davidson**	hateful	1,430	5,593
	not-hateful	4,163

**Table 3:** Distribution of instances in the train/test sets in topic-specific datasets

Topic	Racism (**Waseem**)				Sexism (**Waseem**)		
	Racism		Non-racism	Total	Sexism	Non-sexism	Total
Train	1,346		7,943	9,289	2,253	7,943	10,196
Test	611		3,373	3,984	963	3,373	4,336
Topic	Misogyny (**AMI corpora** + **HatEval**)				Xenophobia (**HatEval**)		
	Misogyny		Non-misogyny	Total	Hateful	Non-hateful	Total
Train	Evalita	1,785	2,215	4,000	1,988	3,012	5,000
	HatEval	1,305	1,396	2,701			
	IberEval	1,568	1,683	3,251			
	Total	4,658	5,294	9,952			
Test	Evalita	460	540	1,000	629	870	1,499
	HatEval	623	849	1,472			
	IberEval	283	443	726			
	Total	1,366	1,832	3,198			

In the next three sections, we show how these datasets have been used to develop models that are able to generalize HS across multiple datasets (cf. Generalizing Hate Speech Phenomena Across Multiple Datasets); transfer knowledge across topics and targets (cf. Multi-target Hate Speech Detection); and leverage emotions to improve multi-target HS detection (cf. Emotion-aware Multi-target Hate Speech Detection). The various forms of bias introduced when building these datasets are discussed in Discussions and Error Analysis, as they may have a strong impact on the multi-target experiments proposed in this paper.

## Generalizing Hate Speech Phenomena Across Multiple Datasets

### Methodology

We aim to answer two main research questions:

- *Are models able to capture common properties of HS and transfer this knowledge from topic-generic datasets to topic-specific datasets?*
- *How do these models compare with ones that are trained on topic-specific datasets?*

To this end, we propose the following two configurations:

- $Top^G \longrightarrow Top^S$: Train on topic-general HS datasets (i.e., **Davidson** and **Founta**)^16^ and test on *all* topic-specific datasets (i.e., **Racism**_**Waseem**_, **Sexism**_**Waseem**_, **Misogyny**_**Evalita**_, **Misogyny**_**IberEval**_, **Misogyny**_**HatEval**_, and **Xenophobia**_**HatEval**_) without splitting them into train/test.
- $Top^S \longrightarrow Top^S$: Train on the combined training sets of all topic-specific datasets (i.e., **Waseem**, **HatEval**, **Evalita**, and **IberEval**) and test on the test set of each topic-specific dataset.

These two configurations are cast as a binary classification task, where the system needs to predict whether a given tweet is hateful (1) or not (0). To this end, we experiment with several performing state of the art models for HS detection. This is a necessary first step in measuring to what extent existing models are capable of transferring knowledge across different HS datasets, be they topic-generic or topic-specific.

### Models

Our models are as follows^17^:

**– Baseline**. This model is straight-forward based on a linear support vector classifier (LSVC). The use of linear kernel is based on [63], who argue that the linear kernel has an advantage for text classification. They observe that text representation features are frequently linearly separable. Hereby, the baseline is an LSVC with unigrams, bigrams, and trigrams TF-IDF.

**– LSTM.** This model uses a LSTM network [59] with an architecture consisting of several layers, starting with an embedding layer representing the input to the LSTM network (128 units), followed by a dense layer (64 units) with ReLU activation function. The final layer consists of a dense layer with sigmoid activation producing the final prediction. In order to get the best possible results, we optimized the batch size (16, 32, 64, 128) and the number of epochs (1-5). We used as input either randomly initialized embeddings (**LSTM**) or FastText^18^ English word vectors with an embedding dimension of 300 [54] pre-trained on Wikipedia and Common Crawl (**LSTM**_**FastText**_). LSTM, a type of Recurrent Neural Network, has already been proven as a robust architecture in HS detection [4].

– **CNN**_**FastTex**t_. This model was inspired by [4, 45]. It uses FastText English word vectors (with the dimension of 300) and three 1D convolutional layers, each one using 100 filters and a stride of 1, but with different window sizes (respectively, 2, 3, and 4) in order to capture different scales of correlation between words, with a ReLU activation function. We further downsample the output of these layers by a 1D max-pooling layer and we feed its output into the final dense layer. All the experiments run for a maximum of 100 epochs, with a patience of 10 and a batch size of 32^19^.

**– ELMo**. This model employs ELMo [103], a deep contextualized word representation, which shows a significant improvement in the study of HS [111]. Since we implement ELMo as a Keras layer^20^, we were able to add more layers after the word embedding layer. The latter is followed by a dense layer (256 units) and a dropout rate of 0.1, before being passed to another dense layer (2 units) with a sigmoid activation function, which produces the final prediction. This architecture is fine-tuned based on the number of epochs (1-15) and batch size (16, 32, 64, and 128), and optimized by using Adam optimizer.^21^

**– BERT**. This model uses the pre-trained BERT model (BERT-Base, Cased), [28] on top of which we added an untrained layer of neurons. We then used the HuggingFace’s PyTorch implementation of BERT [139] that we trained for three epochs with a learning rate of 2e-5 and AdamW optimizer. It is based on [122] where it achieved the best results for the task of abusive language detection.

### Results

#### Results for the $Top^G \longrightarrow Top^S$ Configuration

Table 4 and Table 5 present our results when training, respectively, on **Founta** and **Davidson**. We provide our results in terms of accuracy (*A*), macro-averaged F-score ($F_1$), precision (*P*) and recall (*R*) with the best results in terms of $F_1$ presented in bold.

**Table 4:** Results for $Top^G \longrightarrow Top^S$ configuration when training on **Founta**

Dataset	Baseline				LSTM				LSTM$_{\text {FastText}}$			
	*P*	*R*	$F_1$	*A*	*P*	*R*	$F_1$	*A*	*P*	*R*	$F_1$	*A*
Racism${_\text {Waseem}}$	0.680	0.601	0.638	0.850	0.613	0.533	0.570	0.842	0.666	0.585	0.623	0.846
Sexism${_\text {Waseen}}$	0.555	0.516	0.534	0.760	0.585	0.517	0.549	0.771	0.624	0.543	0.581	0.773
Xenophobia${_\text {HatEval}}$	0.632	0.542	0.583	0.622	0.602	0.507	0.550	0.601	0.589	0.509	0.546	0.601
Misogyny${_\text {Evalita}}$	0.627	0.582	0.603	0.612	0.692	0.634	0.662	0.661	0.679	0.649	**0.664**	0.669
Misogyny${_\text {IberEval}}$	0.622	0.569	0.594	0.592	0.669	0.610	0.638	0.630	0.662	0.625	0.643	0.641
Misogyny${_\text {HatEval}}$	0.615	0.584	0.599	0.615	0.632	0.616	0.624	0.636	0.636	0.631	0.633	0.642
Misogyny${_\text {all}}$	0.645	0.584	0.613	0.616	0.655	0.619	0.636	0.643	0.651	0.632	**0.641**	0.649

Dataset	CNN				BERT				**ELMo**			
	*P*	*R*	$F_1$	*A*	*P*	*R*	$F_1$	*A*	*P*	*R*	$F_1$	*A*
Racism_Waseem_	0.700	0.627	0.661	0.855	0.705	0.742	**0.723**	0.840	0.584	0.568	0.575	0.806
Sexism_Waseem_	0.622	0.563	**0.591**	0.767	0.528	0.501	0.514	0.712	0.543	0.524	0.533	0.736
Xenophobia_HatEval_	0.624	0.517	0.565	0.607	0.651	0.652	**0.651**	0.611	0.581	0.520	0.548	0.604
Misogyny_Evalita_	0.649	0.612	0.629	0.637	0.651	0.659	0.654	0.663	0.635	0.608	0.621	0.630
Misogyny_IberEval_	0.629	0.590	0.609	0.609	0.661	0.639	**0.649**	0.661	0.602	0.571	0.586	0.590
Misogyny_HatEval_	0.609	0.595	0.601	0.616	0.632	0.637	**0.634**	0.639	0.620	0.602	0.610	0.625
Misogyny_all_	0.628	0.615	0.621	0.630	0.643	0.637	0.639	0.647	0.627	0.597	0.612	0.621

**Table 5:** Results for $Top^G \longrightarrow Top^S$ configuration when training on **Davidson**

Dataset	Baseline				ELMO				LSTM			
	*P*	*R*	$F_1$	*A*	*P*	*R*	$F_1$	*A*	*P*	*R*	$F_1$	*A*
Racism_Waseem_	0.585	0.560	0.572	0.814	0.665	0.661	**0.663**	0.833	0.573	0.535	0.553	0.852
Sexism_Waseem_	0.558	0.528	0.542	0.747	0.628	0.586	**0.606**	0.761	0.574	0.526	0.549	0.761
Xenophobia_HatEval_	0.601	0.541	0.569	0.615	0.616	0.544	0.577	0.620	0.604	0.517	0.557	0.605
Misogyny_Evalita_	0.668	0.666	0.667	0.672	0.623	0.624	0.624	0.626	0.680	0.681	**0.680**	0.682
Misogyny_IberEval_	0.638	0.633	0.635	0.639	0.632	0.631	0.631	0.635	0.678	0.676	**0.677**	0.680
Misogyny_HatEval_	0.635	0.636	0.635	0.630	0.621	0.622	0.621	0.619	0.638	0.636	0.637	0.623
Misogyny_all_	0.653	0.654	0.654	0.657	0.623	0.617	0.620	0.628	0.657	0.658	0.657	0.656

Dataset	LSTM${_\text {FastText}}$				CNN${_\text {FastText}}$				BERT			
	*P*	*R*	$F_1$	*A*	*P*	*R*	$F_1$	*A*	*P*	*R*	$F_1$	*A*
Racism_Waseem_	0.613	0.656	0.634	0.775	0.622	0.617	0.619	0.812	0.605	0.561	0.582	0.819
Sexism_Waseem_	0.544	0.540	0.542	0.699	0.586	0.557	0.571	0.744	0.544	0.531	0.537	0.741
Xenophobia_HatEval_	0.635	0.547	0.588	0.624	0.641	0.551	**0.592**	0.628	0.635	0.527	0.575	0.607
Misogyny_Evalita_	0.635	0.620	0.627	0.602	0.652	0.653	0.652	0.652	0.676	0.678	0.677	0.673
Misogyny_IberEval_	0.649	0.635	0.643	0.623	0.653	0.653	0.653	0.654	0.663	0.661	0.662	0.661
Misogyny_HatEval_	0.619	0.593	0.606	0.562	0.659	0.647	**0.652**	0.626	0.639	0.644	0.641	0.624
Misogyny_all_	0.633	0.614	0.623	0.594	0.658	0.657	**0.658**	0.648	0.654	0.654	0.654	0.649

We recall here that we focus on learning topic-generic HS properties and test how neural models are able to extrapolate this information in order to detect topic-specific HS. The results show that **ELMo** outperformed other models in the **Waseem** dataset (**Racism**_**Waseem**_, **Sexism**_**Waseem**_) when trained on **Davidson**. When trained on **Founta**, **CNN**_**FastText**_ obtained the best results for **Sexism**_**Waseem**_ and **BERT **for **Racism**_**Waseem**_. For most of the topic-specific testing datasets (**AMI corpora** in particular), the results are comparable across the two general HS training datasets (**Davidson** and **Founta**), with higher disparities being observed in the **Waseem** results.

#### Results for the $Top^S \longrightarrow Top^S$ Configuration

Table 6 presents the results obtained when focusing on learning topic-specific HS properties by combining all training sets of all datasets. The overall picture of the results shows that our baseline (i.e., **LSVC**) performed quite well when compared to other models: it presents a decrease of anywhere in between 1% and 11% in terms of *F*1 score, when compared to the best-performing models for a specific topic. For most topics, the best results were obtained by **BERT**, with the only exception being for the **Misogyny**_**HatEval**_ dataset, where **ELMo** obtained the best results (with a difference of almost 2% in terms of *F*1 score). We note that **Misogyny**_**HatEval**_ is the only dataset for which **ELMo** achieved good results. For all the other datasets, the results are low, even lower than the baseline^22^. We also note that state of the art models achieved good results for both topics in the **Waseem** dataset, whereas they attain lower results when tested on the xenophobia topic from the **HatEval** dataset. However, our results are similar to the ones obtained by state-of-the-art baselines for **Waseem** (*F*1=0.739 [133]) and **HatEval** (*F*1=0.451 [5])^23^.

**Table 6:** Results for $Top^S\longrightarrow Top^S$ when training on **Waseem**, **HatEval** and **AMI** train sets

Dataset	Baseline				LSTM				LSTM$_{\text {FastText}}$			
	*P*	*R*	$F_1$	*A*	*P*	*R*	$F_1$	*A*	*P*	*R*	$F_1$	*A*
Racism_Waseem_	0.786	0.798	0.792	0.889	0.796	0.765	0.779	0.878	0.783	0.783	0.783	0.887
Sexism_Waseem_	0.815	0.790	0.801	0.868	0.787	0.795	0.791	0.857	0.758	0.807	0.775	0.855
Xenophobia_HatEval_	0.572	0.546	0.470	0.497	0.530	0.560	0.427	0.471	0.546	0.589	0.447	0.488
Misogyny_Evalita_	0.645	0.646	0.645	0.646	0.652	0.652	0.648	0.648	0.661	0.660	0.657	0.658
Misogyny_IberEval_	0.803	0.732	0.742	0.778	0.709	0.754	0.717	0.750	0.739	0.793	0.749	0.779
Misogyny_HatEval_	0.659	0.551	0.421	0.487	0.613	0.688	0.534	0.561	0.564	0.665	0.447	0.502
Misogyny_all_	0.630	0.624	0.601	0.602	0.650	0.654	0.631	0.631	0.636	0.644	0.612	0.614

Dataset	CNN${_\text {FastText}}$				BERT				ELMo			
	*P*	*R*	$F_1$	*A*	*P*	*R*	$F_1$	*A*	*P*	*R*	$F_1$	*A*
Racism_Waseem_	0.764	0.800	0.782	0.827	0.775	0.844	**0.802**	0.884	0.616	0.833	0.651	0.874
Sexism_Waseem_	0.793	0.798	0.795	0.816	0.807	0.829	**0.817**	0.869	0.589	0.815	0.599	0.810
Xenophobia_HatEval_	0.492	0.471	0.481	0.462	0.619	0.543	**0.578**	0.577	0.562	0.596	0.543	0.609
Misogyny_Evalita_	0.673	0.684	0.678	0.684	0.704	0.705	**0.704**	0.706	0.562	0.672	0.496	0.594
Misogyny_IberEval_	0.713	0.742	0.727	0.735	0.841	0.840	**0.840**	0.848	0.538	0.774	0.460	0.639
Misogyny_HatEval_	0.603	0.532	0.565	0.553	0.694	0.523	0.596	0.573	0.618	0.643	**0.615**	0.649
Misogyny_all_	0.671	0.640	0.655	0.651	0.703	0.697	**0.676**	0.677	0.583	0.646	0.557	0.630

In order to assess whether training on topic-specific data improves the results beyond those achieved by training on topic-generic data, we compare our results with both the baselines and the best-submitted systems in the shared task competition where these data have been used (only available for **AMI corpora**). The comparison was made by training either on a topic-general dataset (i.e., $Top^G \longrightarrow Top^S$) or on all topic-specific datasets (i.e., $Top^S \longrightarrow Top^S$), and testing the test data provided by the organizers of AMI-IberEval and AMI-Evalita. Table 7 shows our results.

**Table 7:** Comparison with related work in terms of accuracy

System	Misogyny_Evalita_	Misogyny_IberEval_
	*A*	*A*
Competition Baseline	0.605	0.783
Competition Best System	0.704	**0.913**
Best$Top^G ({{Founta}} ) \longrightarrow Top^S$(**ELMo**/**BERT**)	0.597	0.697
Best$Top^G ({{Davidson}}) \longrightarrow Top^S$(**BERT**/**ELMo**)	0.610	0.658
Best$Top^S (all) \longrightarrow Top^S$(**BERT**)	**0.706**	0.848

When compared to the **AMI Misogyny**_**Evalita**_ and **Misogyny**_**IberEval**_ baselines^24^ provided in terms of accuracy (respectively, 0.605 and 0.783), we observe that using a topic-specific training approach, **BERT** achieved more than a 10% increase for both datasets, while for the topic-generic training approach the only improvement of (0.5%) is brought by **BERT** trained on the **Davidson** dataset (for **Misogyny**_**Evalita**_). When comparing the results with the best-submitted systems (0.704 and 0.913^25^) we still observe a small improvement achieved by **BERT** trained on topic-specific data for the **Misogyny**_**Evalita**_ task, though all the other system results were lower. These results confirm that a model trained with a combination of several datasets with different topical focuses is more robust than a model trained on a topic-generic dataset.

## Multi-target Hate Speech Detection

### Methodology

Now that we have established that the topic-generic datasets are not adequate for capturing specific instances of HS using state of the art HS detection models, the next step is to evaluate how topically focused datasets can be used to detect multi-target HS. This implies answering two main research questions:

- *Is combining topic-specific datasets better for predicting HS towards a given seen topic/target?*
- *What happens when the models are tested on a topic-specific dataset where the topic and/or the target are unseen?*

Let *T* be either a topic (*Top*) or a target (*Tag*). We propose the following configurations:

- $T^S \longrightarrow T_{seen}^S$: We model the task as a multi-label classification problem with two sub-configurations:
  - $Top^S \longrightarrow Top_{seen}^S$: Detect the hatefulness of a given tweet and the topic to which the HS belongs. Each tweet is thus classified into eight different classes, representing the combination of the four topics (racism, sexism, misogyny, xenophobia) and two HS classes (hate speech vs. non hate speech). As in the previous experiments (cf. Methodology), we combine all the training sets of the topic-specific datasets for training. Then, all the models are tested on the test set of each topic-specific datasets.
  - $Tag^S \longrightarrow Tag_{seen}^S$: It is similar to (a), except that it concerns the multi-label classification of targets. Therefore, we merge topic-specific train and test sets that share the same target (i.e. *women*: **Sexism**_**Waseem**_ and **Misogyny**_**all**_ and *ethnicity*: **Racism**_**Waseem**_ and **Xenophobia**_**HatEval**_).
- $T^S \longrightarrow T_{unseen}^S$: We model the task as a binary classification task to predict the topic/target not previously seen during training time. We also design two experiments here:
  (iii) $Top^S \longrightarrow Top_{unseen}^S$: It uses three out of the four topic datasets for training and the remaining topic dataset for testing (i.e., the dataset left out at training time). For example, to detect the hatefulness of misogynistic messages, we train on the following topics: racism (**Racism**_**Waseem**_), sexism (**Sexism**_**Waseem**_) and xenophobia (**Xenophobia**_**HatEval**_), then we test on the misogyny topic (i.e., comprising **AMI corpora** and **Misogyny**_**HatEval**_).
  (iv) $Tag^S \longrightarrow Tag_{unseen}^S$: It is similar to (c), except that it concerns targets. For example, to detect the hateful messages that target women, we train by using the datasets related to the target race (i.e., **Racism**_**Waseem**_ and **Xenophobia**_**HatEval**_) and test on the four datasets related to the target *women* (i.e., **Sexism**_**Waseem**_, the two **AMI corpora** and **Misogyny**_**HatEval**_).

Both $T^S \longrightarrow T_{seen}^S$ (multi-label classification) and $T^S \longrightarrow T_{unseen}^S$ (binary classification) rely on the six models presented in Methodology (i.e., **LSVC**, **LSTM**, **LSTM**_**FastText**_, **CNN**_**FastText**_, **ELMo**, and **BERT**). In addition, for $T^S \longrightarrow T_{seen}^S$we propose a multi-task setting that consists of two classifiers that are trained jointly by multi-task objectives. The first classifier predicts whether the tweet is hateful or not (0 and 1), while the second one the topic of HS (racism (0), sexism (1), misogyny (2), and xenophobia (3)). The final label prediction is broken down into eight classes (cf. Table 8). The multi-task systems are compared to the previous six models used here as strong baselines.

**Table 8:** Label combination in multi-task setting

Target Label	Hate Speech Label	Final Label
Racism (0)	Not Hate Speech (0)	Not Racism (0)
	Hate Speech (1)	Racism (1)
Sexism (1)	Not Hate Speech (0)	Not Sexism (2)
	Hate Speech (1)	Sexism (3)
Misogyny (2)	Not Hate Speech (0)	Not Misogyny (4)
	Hate Speech (1)	Misogyny (5)
Xenophobia (3)	Not Hate Speech (0)	Not Hate Speech towards immigrants (6)
	Hate Speech (1)	Hate Speech towards immigrants (7)

MTL has already been successfully applied in cross-domain aspect-based sentiment analysis (cf. Affective Computing and Sentiment Analysis and Domain Adaptation in Abusive Language Detection for related work in the field) and is used here for the first time in an HS detection task, making a parallel between the sentiment domain (e.g., restaurant, book, hotel, etc.) and the topic/target of HS. Indeed, the main problem in sentiment analysis is the big performance decline in the out-domain setting (when a system is trained and tested with different dataset domains) compared to the in-domain setting (when a system is trained and tested on dataset within the same domain). Similar challenges also arise in the abusive language detection task, where a system is struggling to obtain a robust performance when trained and tested with different datasets. These usually have different focuses on the phenomena they want to capture.

### Models

We experiment with state of the art models (i.e., **LSVC**, **LSTM**, **LSTM**_**FastText**_, **CNN**_**FastText**_, **ELMo**, and **BERT**, as described in Models) and extend them with a multi-task architecture, as described below:

–**LSTM**_**multi-task**_. First, we investigate successful approaches in multi-domain sentiment analysis, a research area that is more mature in dealing with multi-domain classification. For example, [74] used Bi-LSTM networks with adversarial training [46, 53] for learning general representation from all domains data. [102] proposed a co-training approach for jointly learning the representation from both domain-invariant and domain-specific representations, while [12, 146] adopted a MTL approach. Among existing models, we decided to re-implement the system proposed in [12], as it has been shown to outperform existing models in one of the most used multi-domain sentiment classification benchmark dataset [73]. This system consists of two Bi-LSTM classifiers, each of them classifying the domain (domain classifier) and the sentiment (sentiment classifier) of the tweets at the same time, with the loss of both tasks being added up. The output of the Bi-LSTM domain classifier is concatenated to the word embedding layer of the sentiment classifier to acquire a domain-aware representation. Then, the output of average pooling (after Bi-LSTMs) of the domain classifier is also concatenated to the sentiment classifier to obtain domain-aware attention.

We extend the architecture proposed in [12]. The first Bi-LSTM predicts whether a given tweet is hateful or not, while the second one predicts the topic/target of HS. In this way, we obtain both topic/target-aware representation and topic/target-aware attention when predicting whether the tweet is hateful or not. For experiments, we fine-tune this model by varying the number of epochs (1-15) and batch-sizes (16, 32, 64, and 128) while keeping the same configurations as in [12]. The model input is either embeddings randomly initialized (**LSTM**_**multi-task**_) or FastText pre-trained embeddings, (**LSTM**_**multi-task (FastText)**_)^26^.

–**ELMo**_**multi-task**_. We also modify our **ELMo **system (cf. Methodology) in order to be able to use it in multi-task setting. Therefore, we built two ELMo-based architectures to predict the hatefulness and topic/target of tweets. Each architecture starts with the ELMo embedding layer, followed by a dense layer with a ReLU activation function, before being passed into another dense layer with a sigmoid activation function to produce the final prediction. Since ELMo embeddings are not trainable, we could not get the topic/target-aware representation as in the previous Bi-LSTMs model. We can only transfer knowledge by concatenating the output of the first dense layer of the topic/target classifier to the dense layer of the hateful classifier. In this way, we expect to get meaningful information about the topic/target to classify the hatefulness of tweets. Again, we only tune the systems by optimizing the number of epochs and batch-sizes.

–**BERT**_**multi-task**_. This model is similar to [75], where all tasks share and update the same low layers (i.e., **BERT** layers), except for the task-specific classification layer. In this architecture, after transferring the text to contextual embeddings in the shared layers and retrieving the first token hidden state of the shared **BERT** model, we apply a dropout of 0.1 and connect it to two different layers (corresponding to the two classification tasks: topic/target and hatefulness). To preserve individual task-specific loss functions and to perform training at the same time, we defined the losses for the two tasks separately and optimized them jointly (by backpropagating their sum through the model). This model was trained for three epochs with a learning rate of 2e-5 and AdamW optimizer.

### Results

#### Results for the $T^S \longrightarrow T_{seen}^S$ Configurations

Table 9 and Table 10 present the results obtained in the $Top^S \longrightarrow Top_{seen}^S$ configuration in which the testing topic was previously seen during training. Table 9 presents the baseline results while Table 10

the multi-task results. We can observe that multi-task models are the best, outperforming all the baselines, the best systems being **LSTM**_**multi-task (FastText)**_ and **BERT**_**multi-task**_. The results obtained on the **Waseem** dataset surpass all the others, which could be a consequence of the higher number of instances in this particular dataset when compared to the others. Overall, the best performance for the multi-topic HS detection task is achieved by **BERT**_**multi-task**_, which attains the best result in eight out of nine test datasets.

**Table 9:** Baseline results for $Top^S \longrightarrow Top_{seen}^S$

Dataset	Baseline				LSTM				LSTM$_{\text {FastText}}$			
	*P*	*R*	$F_1$	*A*	*P*	*R*	$F_1$	*A*	*P*	*R*	$F_1$	*A*
Racism_Waseem_	0.701	0.844	0.766	0.610	0.841	0.827	0.834	0.856	0.816	0.856	**0.835**	0.855
Sexism_Waseem_	0.694	0.852	0.765	0.545	0.781	0.859	0.818	0.827	0.782	0.869	**0.826**	0.832
Xenophobia_HatEval_	0.474	0.544	0.507	0.404	0.459	0.601	0.521	0.387	0.496	0.651	0.563	0.421
Misogyny_Evalita_	0.614	0.653	0.633	0.612	0.598	0.657	0.626	0.599	0.609	0.661	0.634	0.604
Misogyny_IberEval_	0.642	0.841	0.728	0.643	0.504	0.716	0.592	0.502	0.607	0.782	0.684	0.582
Misogyny_HatEval_	0.518	0.578	0.546	0.452	0.595	0.644	0.618	0.551	0.536	0.662	0.592	0.468
Misogyny_all_	0.576	0.638	0.605	0.545	0.574	0.638	0.604	0.555	0.573	0.645	0.607	0.536

Dataset	CNN${_\text {FastText}}$				BERT				ELMo			
	*P*	*R*	$F_1$	*A*	*P*	*R*	$F_1$	*A*	*P*	*R*	$F_1$	*A*
Racism_Waseem_	0.703	0.754	0.727	0.855	0.847	0.597	0.701	0.791	0.819	0.840	0.829	0.859
Sexism_Waseem_	0.841	0.810	0.825	0.826	0.876	0.666	0.757	0.812	0.675	0.854	0.754	0.788
Xenophobia_HatEval_	0.532	0.491	0.510	0.422	0.667	0.527	**0.588**	0.516	0.356	0.567	0.437	0.312
Misogyny_Evalita_	0.653	0.586	0.618	0.595	0.723	0.672	**0.697**	0.670	0.427	0.650	0.516	0.431
Misogyny_IberEval_	0.865	0.725	0.788	0.724	0.857	0.783	**0.818**	0.780	0.484	0.738	0.585	0.531
Misogyny_HatEval_	0.602	0.563	0.582	0.505	0.681	0.581	**0.627**	0.632	0.529	0.624	0.573	0.488
Misogyny_all_	0.656	0.612	0.633	0.643	0.702	0.654	**0.677**	0.657	0.488	0.634	0.551	0.479

**Table 10:** Multi-task results for $Top^S \longrightarrow Top_{seen}^S$

Dataset	LSTM$_{\text {multi-task}}$				LSTM$_{\text {multi-task(FastText)}}$			
	*P*	*R*	$F_1$	*A*	*P*	*R*	$F_1$	*A*
Racism_Waseem_	0.787	0.851	0.818	0.877	0.839	0.811	0.825	0.828
Sexism_Waseem_	0.774	0.867	**0.818**	0.848	0.763	0.842	0.801	0.797
Xenophobia_HatEval_	0.475	0.534	0.503	0.407	0.495	0.621	0.551	0.422
Misogyny_Evalita_	0.573	0.639	0.604	0.560	0.621	0.687	**0.653**	0.605
Misogyny_IberEval_	0.556	0.774	**0.647**	0.542	0.644	0.792	**0.710**	0.621
Misogyny_HatEval_	0.551	0.650	0.597	0.489	0.554	0.682	**0.612**	0.489
Misogyny_all_	0.560	0.651	0.602	0.523	0.597	0.684	**0.637**	0.555

Dataset	ELMO$_{\text {multi-task}}$				BERT$_{\text {multi-task}}$			
	*P*	*R*	$F_1$	*A*	*P*	*R*	$F_1$	*A*
Racism_Waseem_	0.677	0.862	0.758	0.827	0.835	0.667	**0.742**	0.865
Sexism_Waseem_	0.599	0.862	0.707	0.764	0.870	0.703	**0.777**	0.874
Xenophobia_HatEval_	0.356	0.617	**0.451**	0.340	0.650	0.585	**0.616**	0.513
Misogyny_Evalita_	0.457	0.594	**0.517**	0.472	0.725	0.685	**0.704**	0.684
Misogyny_IberEval_	0.479	0.714	0.573	0.541	0.865	0.774	0.817	0.774
Misogyny_HatEval_	0.580	0.615	**0.597**	0.580	0.701	0.598	**0.646**	0.642
Misogyny_all_	0.520	0.613	**0.563**	0.538	0.721	0.648	**0.682**	0.683

Table 11 presents the results obtained for the $Tag^S \longrightarrow Tag_{seen}^S$ experiments in which the testing target was previously seen during training. The best result for the target women was obtained by **CNN**_**FastText**_, while for the target race **LSTM**_**multi-task (FastText)**_ outperformed all the other models. Our results confirm our assumption that the multi-task approach is capable of a robust performance in a multi-topic experiment, proving its ability in transferring knowledge between different topics, as reported in previous cross-domain sentiment analysis studies.

**Table 11:** Baselines and multi-task results for$Tag^S \longrightarrow Tag_{seen}^S$

System	women				ethnicity			
	*P*	*R*	$F_1$	*A*	*P*	*R*	$F_1$	*A*
LSVC	0.530	0.704	0.605	0.431	0.548	0.632	0.587	0.457
LSTM	0.678	0.713	0.695	0.711	0.650	0.608	0.628	0.728
LSTM_FastText_	0.677	0.721	0.698	0.707	0.656	0.621	0.638	0.737
CNN_FastText_	0.732	0.716	**0.724**	0.731	0.580	0.435	0.497	0.613
BERT	0.772	0.660	0.712	0.681	0.652	0.638	0.645	0.651
ELMo	0.582	0.654	0.616	0.657	0.588	0.656	0.620	0.710
LSTM_multi-task_	0.667	0.719	0.692	0.710	0.631	0.649	0.640	0.774
LSTM_multi-task (FastText)_	0.680	0.725	0.701	0.694	0.667	0.673	**0.670**	0.717
ELMo_multi-task_	0.559	0.678	0.613	0.668	0.516	0.694	0.592	0.694
BERT_multi-task_	0.772	0.671	0.718	0.692	0.649	0.642	0.645	0.657

#### Results for the $T^S \longrightarrow T_{unseen}^S$ Configuration

We begin by presenting the results in the $Top^S \longrightarrow Top_{unseen}^S$ experiments in which the testing topic was unseen during training. As shown in Table 12, we observe that in the absence of data annotated for a specific type of HS, one can use (already existing) annotated data for different kinds of HS.

As this experiment is cast as a binary classification task, we compare the results with the ones presented in Table 6 that concern $Top^S \longrightarrow Top^S$ when training on **Waseem**, **HatEval** and **AMI** train sets and where topics are seen in the test sets. We noticed that **CNN**_**FastText**_ was able to achieve a similar performance for the topic misogyny (0.655 in both $Top^S \longrightarrow Top_{unseen}^S$ and $Top^S \longrightarrow Top^S)$, improving almost 2% for the target xenophobia (moving from 0.578 in $Top^S \longrightarrow Top^S$ with **BERT **to 0.595 in terms of $F_1$). However, lower results were obtained for the Waseem dataset, where the drop in terms of $F_1$ is between 15% and 20%. The overall results also show that **CNN**_**FastText**_ was the best in predicting unseen topics for the four topics we experiment on. By capturing different scales of correlation between words (i.e., bigrams, trigrams, and unigrams), the CNN model can detect different patterns in the sentence, regardless of their position [116].

Finally, Table 13 presents the results obtained when the models are trained on all the available data belonging to a target and tested on all the available data belonging to a different target (i.e., $Tag^S \longrightarrow Tag_{unseen}^S$). In line with the previous experiment, the best results were achieved by **CNN**_**FastText**_. In order to better interpret these results, we conducted another experiment in which a model is trained only on data belonging to a target and tested on data belonging to a topical focus on a different target (e.g., training on the target women and testing on the topic xenophobia belonging to the target race). When comparing these results (cf. Table 14) with the ones presented in Table 12, one can observe the importance for the system of having learned some information regarding the target, even if the data belong to a different topical focus. In the absence of such information, a drop of anywhere in between 1% and 12% can be observed for the best-performing models.

**Table 12:** Results for $Top^S \longrightarrow Top_{unseen}^S$.

System	Racism_Waseem_				Sexism_Waseem_			
	*P*	*R*	$F_1$	*A*	*P*	*R*	$F_1$	*A*
LSVC	0.458	0.490	0.474	0.820	0.491	0.498	0.494	0.761
LSTM	0.481	0.462	0.471	0.790	0.525	0.543	0.534	0.731
LSTM_FastText_	0.489	0.460	0.473	0.787	0.507	0.518	0.513	0.740
ELMo	0.492	0.489	0.491	0.769	0.502	0.506	0.504	0.745
CNN_FastText_	0.742	0.506	**0.602**	0.853	0.882	0.545	**0.674**	0.798
BERT	0.507	0.500	0.504	0.842	0.693	0.537	0.605	0.785

System	Misogyny_all_				Xenophobia_HatEval_			
	*P*	*R*	$F_1$	*A*	*P*	*R*	$F_1$	*A*
LSVC	0.580	0.581	0.581	0.577	0.629	0.536	0.579	0.603
LSTM	0.562	0.563	0.562	0.545	0.541	0.557	0.549	0.583
LSTM_FastText_	0.564	0.572	0.568	0.535	0.508	0.560	0.535	0.583
ELMo	0.510	0.556	0.532	0.583	0.511	0.542	0.526	0.573
CNN_FastText_	0.659	0.652	**0.655**	0.638	0.598	0.593	**0.595**	0.617
BERT	0.634	0.628	0.631	0.639	0.617	0.531	0.571	0.614

**Table 13:** Results for $Tag^S \longrightarrow Tag_{unseen}^S$

System	women				ethnicity			
	*P*	*R*	$F_1$	*A*	*P*	*R*	$F_1$	*A*
LSVC	0.399	0.491	0.440	0.676	0.438	0.491	0.463	0.753
LSTM	0.423	0.489	0.453	0.670	0.500	0.500	0.500	0.744
LSTM_FastText_	0.445	0.487	0.465	0.659	0.476	0.489	0.482	0.722
ELMo	0.420	0.486	0.451	0.665	0.437	0.486	0.460	0.743
CNN_FastText_	0.579	0.513	**0.544**	0.660	0.665	0.543	**0.598**	0.773
BERT	0.514	0.501	0.507	0.656	0.596	0.506	0.548	0.766

**Table 14:** Results for $Tag^S \longrightarrow Top_{unseen}^S$

System	Train on target: ethnicity and test on:							
	Racism_Waseem_				Xenophobia_HatEval_			
	*P*	*R*	$F_1$	*A*	*P*	*R*	$F_1$	*A*
LSVC	0.446	0.488	0.466	0.819	0.494	0.499	0.497	0.577
LSTM	0.432	0.478	0.451	0.805	0.469	0.486	0.478	0.548
LSTM_FastText_	0.434	0.475	0.451	0.798	0.480	0.492	0.486	0.557
ELMo	0.445	0.481	0.462	0.805	0.510	0.501	0.505	0.577
CNN_FastText_	0.716	0.504	**0.592**	0.852	0.563	0.534	**0.548**	0.600
BERT	0.553	0.502	0.526	0.849	0.547	0.505	0.525	0.597

System	Train on target: ethnicity and test on:							
	Sexism_Waseem_				Misogyny_all_			
	*P*	*R*	$F_1$	*A*	*P*	*R*	$F_1$	*A*
LSVC	0.391	0.486	0.431	0.756	0.498	0.470	0.484	0.569
LSTM	0.395	0.484	0.431	0.753	0.500	0.500	0.500	0.571
LSTM_FastText_	0.403	0.479	0.431	0.741	0.474	0.495	0.484	0.560
ELMo	0.419	0.479	0.436	0.737	0.452	0.495	0.472	0.565
CNN_FastText_	0.843	0.504	**0.631**	0.780	0.576	0.532	**0.553**	0.570
BERT	0.446	0.498	0.470	0.774	0.483	0.498	0.490	0.546

To conclude, the results confirm that the multi-task approach is able to achieve a robust performance, especially for the multi-topic HS detection task. These results are encouraging as they can constitute the first step towards targeted HS detection. This would be especially true for languages that lack annotated data for a particular target or in the aftermath of a triggering event.

## Emotion-aware Multi-target Hate Speech Detection

### Methodology

In this section, we focus on investigating the following questions:

- *To what extent does injecting domain-independent affective knowledge encoded in sentic computing resources and in semantically structured hate lexicons improve the performance for the two finer-grained tasks (i.e., detecting the hatefulness of a tweet and its topical focus)?*
- *Which emotional categories are the most productive?*

We experiment with several affective resources that have been proven useful for tasks related to sentiment analysis, including abusive language detection (cf. Affective Information in Abusive Language Detection Tasks). Psychological studies suggest that abusive language is often deeply linked to the emotional state of the speaker, and that this is reflected in the affective characteristics of the haters’ language. Our intuition, then, was that it would be reasonable to inject knowledge about emotions into our models as a domain-independent signal that might help to detect HS at a finer-grained level of granularity across different topical focuses and targets. In particular, we rely on:

- two concept-level resources from the sentic computing framework, where affective knowledge about basic and complex emotions is encoded, concerning different psychological models of emotions: SenticNet^27^ [18] and EmoSenticNet^28^ [106], where emotional labels are related to the Plutchik [104] and Ekman’s [31] models of emotions.
- a hate lexicon (Hurtlex), where lexical information is structured in different categories depending on the nature of the hate expressed, to see whether this multifaceted affective information, specifically related to the hate domain, helps multi-topic and multi-target detection.

As discussed in Related Work, emotion features have already been used in several NLP tasks (e.g., sentiment analysis [95] and figurative language detection [35, 120]). However, to the best of our knowledge, no one has investigated the impact of emotion features on HS detection. In particular, we make use of several affective resources (HurtLex and, for the first time, Sentic resources) and identify the emotion categories that are the most productive in detecting HS towards a given topic/target. To this end, we designed the following two experiments (we recall that *T* refers either to a topic (*Top*) or a target (*Tag*)):

- ($T^S \longrightarrow T_{seen}^S )^{Hurt}$ and ($T^S \longrightarrow T_{seen}^S )^{Sentic}$ where we, respectively, add features extracted from HurtLex and SenticNet (both from SenticNet and EmoSenticNet) on top of the models presented in Methodology and Methodology.
- ($Top^S \longrightarrow Top_{unseen}^S )^{Sentic}$ where we explore the impact of general affect lexica on topically focused datasets.

The models developed for each experiment are detailed below.

### Models

#### Sentic-based Models

SenticNet consists of a collection of commonly used concepts with polarity (i.e., commonsense concepts with relatively strong positive or negative polarity), where each concept is associated with emotion categorization values expressed in terms of the Hourglass of emotions model [16], which organizes and blends 24 emotional categories from Plutchik’s model into four affective dimensions (*pleasantness*, *attention*, *sensitivity*, and *aptitude*). Each of these four dimensions is characterized by six *sentic levels* that measure the strength of an emotion. In this paper, we use SenticNet 5 that includes over 100,000 natural language concepts.

EmoSenticNet is another concept-based lexical resource and was automatically built by merging WordNet-Affect [119] and SenticNet, with the main aim of having a complete resource containing not only quantitative polarity scores associated with each SenticNet concept but also qualitative affective labels  [106]. In particular, it assigns WordNet-Affect emotion labels related to Ekman’s six basic emotions (disgust, sadness, anger, joy, fear, and surprise) to SenticNet concepts. The whole list currently includes 13,189 annotated entries.

Several approaches for representing the affective information included in these two resources were tested by creating feature vectors composed of:

- 24 basic emotions extracted from SenticNet (six basic emotions for each of the four dimensions);
- 16 second level emotions extracted from SenticNet (these emotions are the result of combining the ‘sentic levels’ pairwise)
- all the affective information extracted from SenticNet (i.e., basic emotions and second level emotions);
- six emotions extracted from EmoSenticNet
- emotions extracted from both SenticNet and EmoSenticNet;
- 24 basic emotions extracted from SenticNet only for the concepts present in Hurtlex;

All these additional features are concatenated with the previously described systems (cf. Methodology and Methodology). The concatenation procedure depends on the architecture of the model, as follows:

- For the **LSTM**-based and **CNN** models, we concatenate the feature representation on the dense layer after the **LSTM**/**CNN** network.
- For the **ELMo** model, the feature representation is injected in the dense layer, after the **ELMo** embedding layer.
- After padding the feature vector to a size equal to the **BERT** model input size, these additional features are passed to a linear layer. The output of the features linear layer is then concatenated with the output of the **BERT** model, which will then be treated as input for the final linear layer.

#### Hurtlex-based Models

HurtLex is a multilingual hate word lexicon, which includes a wide inventory of about 1,000 hate words (originally compiled in a manual fashion for Italian by the linguist Tullio De Mauro [27]^29^) organized into 17 categories grouped in different macro-levels [6]:

- *Negative stereotypes*: ethnic slurs (PS); locations and demonyms (RCI); professions and occupations (PA); physical disabilities and diversity (DDF); cognitive disabilities and diversity (DDP); moral and behavioral defects (DMC); and words related to social and economic disadvantage (IS).
- *Hate words and slurs beyond stereotypes*: plants (OR); animals (AN); male genitalia (ASM); female genitalia (ASF); words related to prostitution (PR); and words related to homosexuality (OM).
- *Other words and insults*: descriptive words with potential negative connotations (QAS); derogatory words (CDS); felonies and words related to crime and immoral behavior (RE); and words related to the seven deadly sins of Christian tradition (SVP).

The lexicon has been translated into over 50 languages (English included) semi-automatically, by extracting all the senses of all the words from BabelNet [93]. We were relying on the English version of Hurlex^30^. Out of the 17 categories, the following were selected for the two vulnerable categories targeted in the four specific manifestations of hate that we address in this paper.

- *misogyny* and *sexism*: male genitalia, female genitalia, words related to prostitution, physical disabilities and diversity, cognitive disabilities and diversity
- *xenophobia* and *racism*: animals, felonies and words related to crime and immoral behavior, ethnic slurs, moral and behavioral defects

We included this specific selection of the HurtLex categories features since a preliminary manual inspection of hateful contents targeting the two vulnerable groups suggests that different subsets of the HurtLex categories can be relevant in detecting any hateful speech against those targets. Moreover, concerning misogyny, we already have some positive experimental evidence about this selection from previous exploitation of Hurtlex for detecting HS targeting women [97, 99].

We experimented with a number of representations of the selected features to train several classifiers:

- each of the selected Hurtlex categories is used as an independent feature (binary or frequency);
- all the selected Hurtlex categories (keeping in mind the choices made for the different targets) are combined in a single feature (i.e., at least one word from at least one of the categories is present) (binary or frequency).

### Results

In the following, we present our results on injecting affective features in our models for all the configurations considered in Multi-target Hate Speech Detection (i.e., $Top^S \longrightarrow Top_{seen}^S$, $Tag^S \longrightarrow Top_{seen}^S$ and $Top^S \longrightarrow Top_{unseen}^S$). In all the tables below, the models for which the results in terms of $F_1$ score outperformed the models without affective features are presented in bold. Moreover, all the tables present an additional column $\Delta$, to highlight the improvements due to the inclusion of the affective features based on Sentic computing resources and Hurtlex. (i.e., $\Delta =$ **Model +**_**AffectiveFeatures**_ **F1 - Model F1**).

#### Results for Sentic computing emotion features

Table 15 presents the results obtained for the multi-label classification task by incorporating the sentic features (as described in the previous section and summarized below)^31^:

1. Basic emotions extracted from SenticNet
2. Basic emotions extracted from SenticNet only for the concepts present in Hurtlex
3. Second level emotions extracted from SenticNet
4. All SenticNet affective information (basic emotions + second level emotions)
5. Emotions extracted from EmoSenticNet
6. Merging the affective information extracted from both SenticNet and EmoSenticNet

**Table 15:** Results for $(Top^S \longrightarrow Top_{seen}^S )^{Sentic}$ and $(Tag^S \longrightarrow Tag_{seen}^S )^{Sentic}$

Dataset	LSTM_multi-task + sentic_					LSTM_multi-task (FastText) + sentic_				
	*P*	*R*	$F_1$	$\Delta$	*A*	*P*	*R*	$F_1$	$\Delta$	*A*
Racism_Waseem_	0.776	0.855	0.814 (1)	- 0.004	0.865	0.834	0.838	**0.836** (5)	+ 0.011	0.855
Sexism_Waseem_	0.771	0.882	**0.823** (6)	+ 0.005	0.851	0.792	0.854	**0.822** (5)	+ 0.015	0.832
Xenophobia_HatEval_	0.459	0.500	0.479 (5)	- 0.024	0.398	0.504	0.575	0.537 (6)	- 0.014	0.435
Misogyny_Evalita_	0.605	0.682	**0.641** (6)	+ 0.037	0.593	0.599	0.682	0.638 (5)	- 0.015	0.581
Misogyny_IberEval_	0.573	0.752	**0.650** (6)	+ 0.003	0.562	0.639	0.815	**0.716** (5)	+ 0.006	0.615
Misogyny_HatEval_	0.581	0.656	**0.616** (5)	+ 0.019	0.527	0.561	0.670	0.611 (6)	- 0.001	0.499
Misogyny_all_	0.586	0.666	**0.624** (6)	+ 0.022	0.553	0.579	0.680	0.626 (5)	- 0.011	0.514
Racism + Xenophobia	0.616	0.620	0.618 (6)	- 0.022	0.741	0.659	0.656	0.658 (5)	- 0.012	0.734
Sexism + Misogyny	0.679	0.742	**0.709** (6)	+ 0.017	0.725	0.686	0.731	**0.707** (5)	+ 0.006	0.706

Dataset	ELMo_multi-task + sentic_					BERT_multi-task + sentic_				
	*P*	*R*	$F_1$	$\Delta$	*A*	*P*	*R*	$F_1$	$\Delta$	*A*
Racism_Waseem_	0.702	0.851	**0.769** (5)	+ 0.011	0.830	0.855	0.666	**0.749** (3)	+ 0.007	0.863
Sexism_Waseem_	0.623	0.867	**0.725** (1)	+ 0.018	0.789	0.870	0.717	**0.786** (6)	+ 0.009	0.798
Xenophobia_HatEval_	0.377	0.604	**0.464** (1)	+ 0.013	0.365	0.617	0.532	0.571(1)	- 0.045	0.468
Misogyny_Evalita_	0.458	0.611	**0.523** (6)	+ 0.006	0.471	0.714	0.664	0.688 (6)	- 0.016	0.661
Misogyny_IberEval_	0.501	0.765	**0.605** (5)	+ 0.032	0.564	0.866	0.766	0.813 (1)	- 0.004	0.771
Misogyny_HatEval_	0.576	0.613	0.594 (5)	- 0.003	0.575	0.705	0.592	0.644 (4)	- 0.002	0.633
Misogyny_all_	0.522	0.612	**0.563** (5)	+ 0.001	0.539	0.705	0.652	0.677 (6)	- 0.005	0.624
Racism + Xenophobia	0.539	0.686	**0.604** (5)	+ 0.012	0.700	0.696	0.594	0.641 (3)	- 0.004	0.676
Sexism + Misogyny	0.572	0.676	**0.619** (5)	+ 0.006	0.671	0.765	0.685	**0.723** (1)	+ 0.005	0.668

As to the different representation strategies and combinations of sentic resources, we observed that the best results were obtained when integrating either the EmoSenticNet emotions, the first level emotions of SenticNet, or merging the SenticNet and EmoSenticNet emotions. In most cases, when including only the second level emotions of SenticNet, we see a drop in the performance of the model. The last results presented in Table 16 concern the ($Top^S \longrightarrow Top_{unseen}^S )^{Sentic}$ setting in which we added sentic features for measuring the impact of general affective knowledge in predicting unseen topics. Three groups of features improve previous models for all the tested topics:

1. Basic emotions extracted from SenticNet.
2. Emotions extracted from EmoSenticNet.
3. Merging the affective information extracted from both SenticNet and EmoSenticNet.

**Table 16:** Results ($$Top^S \longrightarrow Top_{unseen}^S )^{Sentic}$$

System	Racism_Waseem_					Sexism_Waseem_				
	*P*	*R*	$F_1$	$\Delta$	*A*	*P*	*R*	$F_1$	$\Delta$	*A*
LSTM_sentic_	0.486	0.467	**0.476** (2)	+ 0.005	0.799	0.525	0.541	0.533 (2)	- 0.001	0.727
LSTM_FastText + sentic_	0.495	0.482	**0.488** (3)	+ 0.004	0.818	0.510	0.530	**0.520** (2)	+ 0.007	0.748
ELMo_sentic_	0.499	0.499	**0.499** (1)	+ 0.008	0.771	0.502	0.508	**0.505** (2)	+ 0.001	0.745
CNN_FastText + sentic_	0.751	0.514	**0.610** (1)	+ 0.008	0.854	0.885	0.539	0.670 (2)	- 0.004	0.794
System	Misogyny_all_					Xenophobia_HatEval_				
	*P*	*R*	$F_1$	$\Delta$	*A*	*P*	*R*	$F_1$	$\Delta$	*A*
LSTM_sentic_	0.558	0.584	**0.571** (1)	+ 0.009	0.603	0.567	0.567	**0.567** (1)	+ 0.018	0.554
LSTM_FastText + sentic_	0.542	0.569	0.555 (2)	- 0.013	0.592	0.593	0.592	**0.593 **(1)	+ 0.060	0.588
ELMo_sentic_	0.516	0.574	**0.543** (1)	+ 0.011	0.587	0.511	0.538	0.524 (2)	- 0.002	0.572
CNN_FastText + sentic_	0.660	0.654	**0.657** (1)	+ 0.002	0.640	0.596	0.598	**0.597** (2)	+ 0.002	0.617

#### Results for Hurtlex emotion features

Table 17 reports the results achieved by the best performing models for the $Top^S \longrightarrow Top_{seen}^S$ experiment (cf. Table 9) (i.e., **BERT**_**multi-task**_ and **CNN**_**FastText**_) when incorporating the following most productive Hurtlex features:

1. Hurtlex categories used as binary independent features.
2. Hurtlex categories used as independent features (count).
3. Single binary feature incorporating the selected Hurtlex categories.
4. Single feature incorporating the selected Hurtlex categories (count).

**Table 17:** Results for $(Top^S \longrightarrow Top_{seen}^S )^{Hurtlex}$ and $(Tag^S \longrightarrow Tag_{seen}^S )^{Hurtlex}$

Dataset	CNN_FastText + Hurtlex_					BERT_multi-task + Hurtlex_				
	*P*	*R*	$F_1$	$\Delta$	*A*	*P*	*R*	$F_1$	$\Delta$	*A*
Racism_Waseem_	0.863	0.802	**0.831** (4)	+ 0.104	0.863	0.852	0.753	**0.799** (4)	+ 0.057	0.874
Sexism_Waseem_	0.857	0.833	**0.845** (4)	+ 0.020	0.846	0.858	0.660	0.746 (2)	- 0.031	0.692
Xenophobia_HatEval_	0.644	0.509	**0.569** (2)	+ 0.059	0.438	0.649	0.583	0.614 (2)	- 0.002	0.509
Misogyny_all_	0.668	0.618	**0.642** (4)	+ 0.009	0.606	0.734	0.652	**0.690 **(4)	+ 0.008	0.696
Misogyny_Evalita_	0.656	0.615	**0.635** (3)	+ 0.017	0.611	0.738	0.695	**0.716** (4)	+ 0.012	0.693
Misogyny_IberEval_	0.848	0.718	0.778 (1)	- 0.010	0.728	0.879	0.785	**0.829** (1)	+ 0.012	0.782
Misogyny_HatEval_	0.658	0.642	**0.650** (4)	+ 0.068	0.616	0.705	0.613	**0.656** (4)	+ 0.010	0.659
Racism + Xenophobia	0.695	0.641	**0.667** (1)	+ 0.170	0.734	0.711	0.646	**0.677** (4)	+ 0.032	0.672
Sexism + Misogyny	0.741	0.701	0.720 (4)	- 0.004	0.740	0.756	0.653	0.701 (2)	- 0.017	0.643

In Table 17, the models for which the results in terms of $F_1$ surpassed the previous models are presented in bold^32^. We observe that almost all the additional features were productive and outperformed the previous models. The improvements brought by **CNN**_**fastText+HurtLex**_ were higher compared to **BERT**_**multi-task + Hurtlex**_: ranging from anywhere in between 1% and 17% (respectively, **Misogyny**_**all**_, and **Racism** + **Xenophobia**) vs. 1% and 5% (respectively, **Misogyny**_**HatEval**_ and **Racism**_**Waseem**_). The results of this experiment confirm our original assumption that including affective information and making use of specific lexicons leads to significant improvements in $Top^S \longrightarrow Top_{seen}^S$ experiments.

## Discussions and Error Analysis

### Main Conclusions

The main findings of this paper are:

**Conclusion 1: Training on topic-generic datasets generally fails to account for the linguistic properties specific to a given topic.** First, we experimented with several HS datasets with different topical focuses in a binary classification setting. This was done in order to capture general HS properties regardless of the dataset type (i.e., topic-generic or topic-specific). We investigated two experimental scenarios: the first one in which a system was trained on a topic-generic dataset and tested on topic-specific data; and a second one in which a given system was trained on a combination of several topic-specific datasets and tested on topic-specific data. The results show that by training a system on a combination of several (training sets from several) topic-specific datasets the system outperforms a system trained on a single topic-generic dataset. This finding partially confirms the assumption made by [122] according to which merging several abusive language datasets could assist in the detection of abusive language in non-generalizable (unseen) problems.

**Conclusion 2: Combining topically focused datasets enabled the detection of multi-target HS even if the topic and/or target are unseen.** Second, we proposed a classification setting which allows a given system to detect not only the hatefulness of a tweet, but also its topical focus in the context of a multi-label classification approach. Our findings show that a multi-task approach in which the model learns two or more tasks simultaneously, does better, in performance terms, than a single-task system, and the best model is the **BERT**_**multi-task**_. In the same way, we also proposed a cross-topic and cross-target experimental setting for the task of HS detection, where a system is trained on several sets of data with different topical focuses and targets and, then, tested on another dataset where its topical focus and target are unseen during training. Results show that **CNN**_**FastText**_ outperformed all the other systems in all the experimental scenarios. We believe that this is an important finding, which will pave the way for targeted HS manifestations, stimulated by a triggering event and which will solve the problem of a lack of annotated data for a particular topic/target.

**Conclusion 3: Affective knowledge encoded in sentic computing resources and semantically structured hate lexicons improve finer-grained HS detection.** Finally, when injecting domain-independent affective knowledge on top of deep learning architectures, multi-target HS detection improves in both settings where topic/target is seen and unseen at training time. The most useful group of features differ greatly on both topic/target and in terms of the model architectures. In most cases, the models incorporating EmoSenticNet emotions, the first level emotions of SenticNet, a blend of SenticNet and EmoSenticNet emotions or affective features based on Hurtlex, obtained the best results. However, when merging both the affective features based on Hurtlex and sentic computing resources, we observed a decline in the quality of the results.

### Impact of Bias in Multi-target Hate Speech Detection

As observed in [127], HS datasets might contain systematic biases towards certain topics and targets. In the context of automatic content moderation, the danger posed by bias is considerable, as bias can unfairly penalize the groups that the automatic moderation systems were designed to protect.

In line with previous works, we observed that bias has a strong impact on target-based HS detection. Based on the results obtained in the cross-topic (i.e., $Top^S \longrightarrow Top_{unseen}^S$ configuration, cf. Table 12), we noted a big performance drop in both **Racism**_**Waseem**_ and** Sexism**_**Waseem**_ when compared to the $Top^S \longrightarrow Top_{seen}^S$ classification setting, as presented in Table 6. One possible explanation for this drop is the bias problems characterizing the **Waseem** dataset. As shown in [136], the **Waseem** dataset contains both author and topic bias, mostly because of their approach to data sampling. The methodology adopted in [136] for studying this issue was also based on the experience of conducting cross-domain experiments (i.e., training on a dataset different from the one used for testing), in order to make the existing bias in abusive language datasets evident. Their results show that datasets that apply a biased sampling for corpus collection (instances matching query words that are likely to occur in abusive language) contain a high degree of implicit abuse. This might lead to a performance decrease due to the difficulty of learning lexical cues that convey implicit abuse.  [136] illustrated how datasets with a high degree of implicit abuse could be more affected by data bias. They observed that when query words and biased words (i.e., the words having the highest Pointwise Mutual Information towards abusive messages) are removed, the performance is much poorer than originally reported.

We draw the same observations in the $Top^G \longrightarrow Top^S$ experiments (cf. Results for the $Top^G \longrightarrow Top^S$ Configuration), where each model is trained on one of the two topic-generic datasets (i.e., **Founta** and **Davidson**) and tested on the topic-specific datasets. As previously mentioned, when comparing the results obtained in Table 4 and Table 5 with the ones presented in Table 6, the biggest performance drop is observed for the **Waseem** dataset. Again, the sampling biases characterizing that dataset may be a contributing factor.

Finally, let us mention the peculiarity of the results that we obtained for the **HatEval** dataset, especially the *xenophobia* portion; this is the only dataset where we observed a definite increase when training on topic-generic datasets, concerning the performances from training on topic-specific data. This counter-trend outcome needs to be further investigated. If possible, it should be investigated in relation to data sampling strategies adopted for **HatEval**, where training and test data were collected in different time frames [42].

### Error Analysis

In this section, we provide an error analysis focusing on the instances for which the predictions of our best performing model (**BERT**_**multi-task**_) and manual annotation differ. We observe that misclassification is affected by several factors, including the absence of context within the utterance and the use of irony, stereotypes, and metaphors. Another relevant factor is the contextual similarities between the topical focuses in those datasets where the vulnerable category target is basically the same, e.g., *misogyny* and *sexism* (see (16) and (17) below^33^) and *xenophobia* and *racism* (see example (18)). In the examples provided below, we underlined some portions of the text in order to highlight the main source, in our view, of misclassification.

(16) * I don’t see why drinking and driving is such a big deal. Letting women drive is just as hazardous!* (gold label: *misogynistic*, predicted: *sexist*)
(17) * HYSTERICAL woman. Not just woman. And, she didnt say he won.* (gold label: *misogynistic*, predicted: *sexist*)
(18) * A piece at a time. Start by  outlawing new Mosques and stoping Muslim immigration.* (gold label: *racist*, predicted: *xenophobia*)

Misogyny and sexism are closely related notions, and the way in which they are related has been the object of investigation in philosophical literature in the last years [78, 110]. In order to take into account relatedness among those and other HS categories, we will consider, in the future, a strategy for putting fewer penalties for errors in predicting closely related topics.

The use of irony is another important source of error. For example, in (19) the underlying stereotype, implying that there is no place for women as TV sportscasters, leads to the message being classified as *non – sexist*. 

(19) * They have to concentrate in the 2nd half of this half”. Wise words from our female commentator.”* (gold label: *sexist*, predicted: *non-sexist*)

In both (20) and (21) the users express their religious views on Islam. The model is not able to correctly predict that these utterances are racist. Complex inference or logical reasoning is needed to understand their point of views.

(20) * The fact that I have a brain prevents me from accepting Islam.* (gold label: *racist*, predicted: *non-racist*)
(21) * If you don’t want to read a pedo, you have to stop reading the Quran.* (gold label: *racist*, predicted: *non-racist*)

Finally, although in (22) the user reports on a series of events, the model predicts the message as conveying hate towards immigrants, most probably because of the use of the word ‘rapefugee’. This is a self-explanatory and derogatory term used for Muslim refugees entering Europe.

(22) * Westminster terror attack suspect named as ’Sudanese Rapefugee who drove around London looking for targets’ before driving car into cyclists* (gold label: *not-hateful against immigrants*, predicted: *hateful against immigrants*)

## Conclusion and Future Work

This paper investigates, for the first time, HS detection from a multi-target perspective, leveraging existing manually annotated datasets with different topical focuses (including sexism, misogyny, racism, and xenophobia) and different targets (gender, ethnicity, religion, and race). Several neural models have been proposed for transferring specific manifestations of hate across topics and targets, while also exploring multi-task approaches and additional affective knowledge. Our results demonstrate that multi-task architectures are the best-performing models and that emotions encoded in sentic computing sources and hate lexicons are important features for multi-target HS detection. This paper thereby shows that multi-target HS detection from existing datasets is feasible. This is the first step towards HS detection for specific topics/targets when dedicated annotated data are missing.

However, there is still room for improvement in building a robust system able to generalize HS towards different topical focuses and targets. In further work, we want to explore other domain adaptation strategies, such as adversarial training. Adversarial training has been shown to be an effective method of learning representations in cross-domain classification in several tasks, including sentiment analysis and image classification [47, 56, 141].

Another path to explore is the impact of bias in multi-target HS detection. Bias in abusive language datasets is an open problem already observed by several previous studies [25, 92, 101, 136], in which different variants of bias, such as topic bias, author bias, gender and racial bias were explored. As no further investigation on developing an approach in debiasing abusive language datasets has been offered, we also plan to examine this direction in the future in the interests of keeping HS detection fair and compliant.

Concerning the role of affective knowledge in detecting hateful contents, we observed that feeding our multi-label classification models with structured knowledge included in a hate lexicon like Hurtlex, where hate words are categorized according to different semantic areas, boosts the performance of the classifiers. This also suggests possible lines of future work. According to the psychological literature, hate words and, in particular, gendered and racial slurs have evolved to the point that they are used, and perceived, to express negative emotions towards targets, therefore providing important information about the speaker’s emotional state or his or her attitude toward the targeted entity [58], even when they refer to descriptive qualities. We, therefore, think that it could be interesting to investigate the link between hateful language and the negative portions of the multifaceted emotion spectrum covered in sentic computing resources. In particular, we plan to test the effectiveness of the new version of the Hourglass model [121], that provides a better understanding of neutral emotions and their association with other polar emotions and that includes some polar emotions that were previously missing (including self-conscious and moral emotions). The revisited Hourglass model calculates the polarity of a concept with higher accuracy. It also provides a new mechanism for classifying unknown concepts by finding the antithetic emotion of a missing concept and by flipping its polarity. SenticNet 6 [15] actually contains 200,000 words and multiword expressions. We believe it may prove a valuable resource for improving multi-topic and multi-target HS detection.

Finally, though most of the available HS corpora are in English, the problem of hateful speech is not limited to one language. Given language diversity and the enormous amount of social media data produced in different regions of the world, the task of detecting HS from a multi-lingual perspective is also a significant challenge. We, therefore, plan, in future, to explore the possibility of developing language-agnostic models capable of identifying HS in online communication.

## Footnotes

1 See [79] for an interesting lexical, linguistic and psycho-linguistic analysis of hateful accounts on Twitter.

2
https://www.internetlivestats.com/twitter-statistics/

3 In this paper, we do not make any distinction between HS directed towards a person/individual or a group, as done in previous studies [132, 143, 144].

4 For a comprehensive description and an evaluation of the different ways lexicons have been employed in sentiment analysis systems, see [95].

5
https://fasttext.cc/

6
https://code.google.com/archive/p/word2vec/

7 In this case, “multilingual” refers to the fact that two datasets were made available as part of the competition. The submitted systems were trained and tested separately on each language.

8 For more details regarding the collection and annotation of the data, the reader is invited to refer to Datasets.

9 Although in the original paper the authors mention that the dataset consists of 24,802 annotated tweets, we only found this number of instances in the shared GitHub repository: https://github.com/t-davidson/hate-speech-and-offensive-language

10 A multilingual repository, which allows for the identification of HS terms by region: https://hatebase.org

11 Now Figure Eight https://www.figure-eight.com/

12 At the moment of collecting the data, from the original dataset http://ow.ly/BqCf30jqffN we were able to retrieve only 44,898 tweets, though in a recent shared task (https://sites.google.com/view/icwsm2020datachallenge/home) the full dataset was made available.

13 When collecting the data, we were able to retrieve only 16,488 instances (3,216 targeting gender minorities, 1,957 racist and 11,315 that were neither racist nor sexist).

14 The only difference with the original paper appears in the training set of the HatEval dataset as we found duplicate instances (already there in the **AMI corpora**).

15 We recall that these two datasets used the same approach for collecting the data and for annotation guidelines.

16 We only use the *hateful* and *not-hateful* instances, although the data are annotated as *hate speech*, *offensive* and *none* (for the **Davidson** dataset) and annotated as *hate speech*, *abusive*, *normal* and *spam* (for the **Founta** dataset).

17 In an exploratory attempt at finding the best way of representing the data, we included a standard pre-processing step (i.e., URLs and user mentions replacement with replacement tokens, RT removal) as well as emoji replacement with their detailed description [118]. However, the results were inconclusive.

18
https://fasttext.cc/

19 All the hyperparameters were tuned on the validation set (20% of the training dataset), such that the best validation error was produced.

20
https://keras.io/

21 We use the default parameter of Adam optimizer as described in https://www.tensorflow.org/api_docs/python/tf/keras/optimizers/Adam

22 The baseline achieved better results in all datasets, except the topics in the **HatEval** dataset.

23 The baseline for the **Waseem** dataset is a LR coupled with character n-grams and the gender information of the tweet author, while the baseline for the **HatEval** shared task is a straightforward SVM with TF-IDF features.

24 SVM with linear kernel trained on the unigram representation of the tweets.

25 The best-submitted system for the AMI Evalita competition is an LR with a vector representation that concatenates sentence embedding, TF-IDF and average word embeddings, while for the AMI IberEval competition it was an SVM with a combination of structural, stylistic and lexical features.

26 GloVe used in the original paper gives lower results.

27
https://sentic.net

28
https://www.gelbukh.com/emosenticnet/

29 The list of hate words has been included in the Final Report (2017) issued by the “Joe Cox” Committee on intolerance, xenophobia, racism and hate, of the Italian Chamber of Deputies.

30
https://github.com/valeriobasile/hurtlex

31 We only report the results achieved by the multi-task models as they performed better (cf. Table 10).

32 An additional experiment consisted in combining the best Hurtlex feature representation with the best sentic feature representation for each of the datasets. However, the results did not improve.

33 Notice that in these two examples the users also rely on stereotypes: ‘women can’t drive’ and ‘women are hysterical’.
